# New Insights Into the Evolution of Color Terms or an Effect of Saturation?

**DOI:** 10.1177/2041669516662040

**Published:** 2016-09-05

**Authors:** Christoph Witzel

**Affiliations:** Université Paris Descartes, France.

**Keywords:** categorization, color, color naming, cross-cultural comparison, evolution, Hadzane, perception, Sapir-Whorf Hypothesis, saturation

## Abstract

Through their thorough investigation of the Hadza, a nonindustrialized language community in Tanzania, Lindsey and colleagues (2015) developed a new approach to understand the evolution of color terms. In the present commentary, I discuss the possibility that some of their results might be explained by the lacking control of saturation of their color stimuli. The saturation of colors plays an important yet widely neglected role in color naming. The additional analyses presented here suggest that the results on Hadzane color naming could be due to variations in saturation in the stimulus set rather than being evidence for universal constraints on color term evolution.

## Introduction

The origin of color categories has preoccupied many scientific disciplines because it is the prime example for studying the biological, ecological, and cultural factors that relate language to perception (Kay & Regier, 2006). Yet, the origin of color categories remains unknown. Through their thorough investigation of the Hadza, a nonindustrialized language community in Tanzania, Lindsey, Brown, Brainard, and Apicella (2015) developed a new approach to understand the evolution of color terms. They observed a low consensus in color naming across Hadza observers, which they took as evidence for a low evolutionary level of the Hadzane color lexicon. Most importantly, this study discovered a latent pattern of color naming across the speakers of the Hadzane language that reflected the motifs present in the English color lexicon, which is supposed to reflect a high evolutionary stage of color naming. From this, the authors concluded that the evolution of color lexica is led through universal motifs that are progressively discovered in the course of evolution through the communicative activities of the speakers.

The theoretical and mathematical approach of this study is truly intriguing. However, with respect to the observed cross-cultural motifs, it is important to note that this study measured color naming with a set of color stimuli that varied strongly in chroma and saturation. Saturation, chroma, and colorfulness (referred to hereafter as “saturation”) generally refer to the difference of a color from achromatic colors, such as black, white, and gray. Until recently, saturation had been neglected in studies on color naming. Seminal studies (e.g., Regier, Kay, & Cook, 2005) used a selection of standard color chips (Munsell chips) and observed cross-cultural regularities in color naming. This classical set of Munsell chips strongly varies in saturation. Because saturation defines the perceptual saliency of colors and distinguishes chromatic from achromatic color categories, it plays a major role in color categorization. Stimuli with particularly high saturation are prone to be assigned to chromatic categories (e.g., red, orange, yellow, and blue); desaturated stimuli are consistently assigned to achromatic categories. It is the stimuli with low saturation which are most ambiguous in category membership (e.g., Figure 8 in Olkkonen, Witzel, Hansen, & Gegenfurtner, 2010).

In fact, the variation of saturation across the classical set of Munsell chips predicts the cross-cultural regularities in color naming observed in those seminal studies (Witzel, Cinotti, & O’Regan, 2015; Witzel, Maule, & Franklin, under revision). It could have been possible that the variation of saturation in the classical stimulus set reflects an inherent property of the visual system, such as a particular sensitivity to saturation for certain hues. In this case, this property of the visual system would work as a universal perceptual constraint on color naming. However, the variation of saturation in that stimulus set does not reflect a property of the visual system; instead, it is simply a peculiarity of that Munsell stimulus set (Witzel & Franklin, 2014; Witzel et al., under revision).

**Figure 1.**
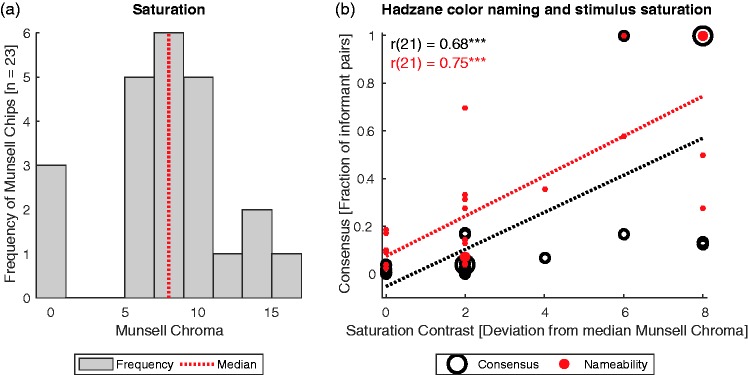
Saturation and color naming of non-industrialized Hadza from Tanzania. Data are taken from Figure 1(e–g) and Table S1 of Lindsey et al. (2015). Panel a shows the frequency of different levels of Munsell chroma among the 23 Munsell chips used by Lindsey et al. (2015). The dotted red line indicates the median Munsell Chroma. Panel b shows the correlation between saturation (Munsell Chroma) and the consensus of Hadza color naming. The *x*-axis shows saturation contrast. The *y*-axis shows the frequency of pairs of Hadza observers who used the same color term to describe the respective color (consensus, black circles), and of observers who used any term (rather than “don't know“) to name the stimuli (nameability, red dots). The size of the circles is scaled by the number of occurrences of the respective data points. The dotted lines show the regression lines. Note the significant correlations, which are robust against outliers (*p* < .001).

In their recent study, Lindsey et al. (2015) sampled stimulus colors from a subset of the classical sample of Munsell chips. The colors in this subset also strongly vary in saturation. This can be seen from the differences in Munsell Chroma, which is an approximate measure of perceived chroma (Figure 1(a)). As a result, some stimuli stand out from the stimulus sample by having either particularly high or particularly low saturation, implying that these colors are more perceptually salient than the other colors. Moreover, in that stimulus set the colors with highest saturation coincide with the prototypes of English color terms, in particular red, orange, yellow, and blue, and the stimuli with lowest saturation are most typical for English black, white, and gray categories. For this reason, an effect of saturation is confounded with patterns of the English color categories.

To more precisely test this possibility, I calculated saturation contrasts as the absolute difference between the Munsell Chroma of each stimulus and the median Munsell Chroma of six (dotted red line in Figure 1(a)). Saturation contrast indicates how much the stimuli stand out due to either a particularly high or particularly low saturation. Lindsey et al. (2015) measured the ability of Hadzane speakers to name and to consistently communicate about colors. This was done by determining the frequency of observer pairs that agreed in the color naming of a stimulus color (*consensus*), and the frequency of observer pairs that produced any color term rather than answering “Don’t know” (*nameability*; cf. blue and green bars in Figure 1(e) of Lindsey et al., 2015, respectively).

Results of the correlational analyses are illustrated in Figure 1(b). Saturation contrasts were positively correlated with both measures in Hadzane speakers (*r*(21) = 0.68 and *r*(21) = 0.74, both *p* < .001). With a toolbox for robust correlation analyses (Pernet, Wilcox, & Rousselet, 2012), I showed that both correlations were statistically robust against outliers (all *p* < .001).

Lindsey and colleagues also report data for Somali and English observers as control groups. Somali observers (Figure 1(f) in Lindsey et al., 2015) also showed positive correlations between saturation and the two color naming measures (consensus and nameability), even though these correlations were less pronounced than for the Hadza (*r* = 0.39 and *r* = 0.41, both *p* = .03 in a one-tailed *t* test). Nameability of English observers was at ceiling (cf. green bars in Figure 1(g) in Lindsey et al., 2015), which prevented the calculation of sensible correlations. Still, the consensus of English observers was also highly correlated with saturation contrast (*r* = 0.57, *p* = .004).

These correlations suggest that the motifs Lindsey and colleagues observed in the Hadzane color lexicon are possibly due to a peculiarity of the stimulus set rather than reflecting universal constraints on color term evolution. This finding highlights the importance of controlling perceptual parameters of stimuli when investigating cognitive phenomena, such as language. This is particularly true for saturation, which has been widely neglected as a dimension of color stimuli in studies on color naming. With respect to the origin of color categories, the question about the evolution of color terms is still open until regularities across languages can be revealed with unbiased stimuli. To exclude the possibility that observed cross-cultural patterns in color categorization are merely due to a peculiarity of the stimulus sample, it is important to investigate these patterns with sets of color stimuli in which the prototypes of English color terms are not particularly saturated and salient.

## Author's Note

The author is currently affiliated to Allgemeine Psychologie, Justus-Liebig Universität, Gießen, Germany.
